# Overexpression of miR164b-resistant *OsNAC2* improves plant architecture and grain yield in rice

**DOI:** 10.1093/jxb/ery017

**Published:** 2018-01-20

**Authors:** Dagang Jiang, Weiting Chen, Jingfang Dong, Jing Li, Fen Yang, Zhichao Wu, Hai Zhou, Wensheng Wang, Chuxiong zhuang

**Affiliations:** 1 State Key Laboratory for Conservation and Utilization of Subtropical Agro-bioresources, College of Life Sciences, South China Agricultural University, Guangzhou, China; 2 Institute of Crop Sciences, Chinese Academy of Agricultural Sciences, Beijing, China

**Keywords:** Architecture, miRNA, *OsNAC2*, rice (*Oryza sativa* L.), yield

## Abstract

Plant architecture is a major target of rice (*Oryza sativa*) breeding and selection, but the underlying regulatory networks remain unclear. Here, we overexpressed an *OsNAC2* mutant (OErN) that cannot be cleaved by the miRNA miR164b. OErN plants had better plant architecture and longer panicles, and produced more grains. The parental line averaged 12.2 primary and 31.5 secondary branches in the main panicles; two OErN lines averaged 15.0 and 15.2 primary, and 41.5 and 44.3 secondary branches. In large-scale field trials, OErN plants produced at least 58.62% more total grain (by weight) compared with the parental line. They also had more large and small vascular bundles in the stem internodes and leaves. Overexpression of miR164b or down-regulation of *OsNAC2* led to decreased panicle length and grain yield in the main panicle. The OErN plants showed significant up-regulation of the grain number and plant architecture-related genes *IPA1* and *DEP1*. A survey of >3000 rice varieties found no natural mutations in the miR164b-binding site of *OsNAC2*. OErN increased yield in Nipponbare and the commonly grown Yangyujing 3 cultivars. In summary, we identified an efficient new strategy to increase rice yield substantially and improve plant architecture through overexpression of OsmiR164b-resistant *OsNAC2*.

## Teaser

Plant architecture affects grain yield. Overexpressing a version of *OsNAC2* resistant to miRNA-mediated degradation increased panicle number and grain yield under field conditions, identifying a new strategy to improve yield.

## Introduction

Increases in population, reductions of available farmland, and environmental deterioration could lead to worldwide food shortages. Therefore, the production of crop varieties with a higher yield per unit area remains of critical importance (McClung, 2014). An estimated 40% increase in rice production will be required by 2030 to support the predicted population growth. Although hybrid rice breeding resulted in a 10–20% increase in grain yield (Khush, 2005), meeting the increasing demand will require new strategies.

Plant architecture encompasses branching pattern, plant height, leaf shape and arrangement, and inflorescence morphology (Reinhardt and Kuhlemeier, 2002; Wang and Li, 2011). Rice breeding strategies for obtaining high-yield varieties have selected for a so-called ideal plant architecture (IPA); these ideotype breeding strategies select for plants that have an optimal combination of low tiller numbers, few unproductive tillers, more grains per panicle (compared with currently cultivated varieties), and thick and sturdy stems (Wang and Li, 2011). Several plant architecture-related genes, corresponding to some yield quantitative loci (QTLs), such as *Gn1a*, *GS3*, *Ghd7*, and *Ghd8*, have been identified (Ashikari *et al.*, 2005; Song *et al.*, 2007; Xue *et al.*, 2008; Yan *et al.*, 2011).

In rice, the *Ideal Plant Architecture 1* gene (*IPA1*) controls the establishment of plant architecture and increases yield potential (Jiao *et al.*, 2010; Miura *et al.*, 2010). *IPA1* encodes SQUAMOSA PROMOTER BINDING PROTEIN-LIKE14 (*OsSPL14*), and IPA1 controls tillering and panicle morphology through the regulation of the downstream gene *DENSE AND ERECT PANICLE1* (*OsDEP1*) (Lu *et al.*, 2013). *IPA1* is regulated at the transcriptional and post-transcriptional levels. For example, a recent study reported that the presence of three tandem repeats upstream of *IPA1*, a naturally occurring variant, can enhance grain yield in super rice varieties such as Yongyou12 (YY12) and related varieties (Zhang *et al.*, 2017). IPA1 INTERACTING PROTEIN 1 (IPI1), a RING-finger E3 ligase that can interact with IPA1 in the nucleus, promotes the degradation of IPA1 in panicles but stabilizes IPA1 in shoot apexes (Wang *et al.*, 2017). In plants with optimal expression of *IPA1*, breeding and selection can then be used to fine-tune other IPA traits; this may lead to improved yield (Zhang *et al.*, 2017).

Ongoing work has shown that miRNAs participate in the regulation of many plant architecture-related genes (Zheng and Qu, 2015). For example, OsmiR156 regulates *IPA1*. OsmiR156 also regulates other *SPL* genes, which control panicle morphology, grain number, and plant architecture (Jiao *et al.*, 2010; Miura *et al.*, 2010; Wang *et al.*, 2012; Si *et al.*, 2016). OsmiR397 influences overall yield by controlling grain number and branches per panicle, via down-regulation of its target gene *OsLAC* (Zhang *et al.*, 2013). OsmiR396b suppresses the expression of growth-regulating factors (GRFs) and regulates GRF/GIF (GRF-interacting factor)-mediated processes in rice growth and development, including grain size, grain number, panicle morphology, and grain yield (Che *et al.*, 2015; Duan *et al.*, 2015; Gao *et al.*, 2015). A natural mutation in *GRAIN SIZE ON CHROMOSOME 2* (*GS2*) perturbs the binding site for OsmiR396c and results in increased grain size and yield (Hu *et al.*, 2015; Li *et al.*, 2016). OsmiR396d regulates spikelet development through regulation of the expression of a spikelet-specific subfamily of *OsGRF* genes, and enhancing the expression of *OsJMJ706* and *OsCR4* (Liu *et al.*, 2014). OsmiR172 regulates the expression of *APETALA2* (*AP2*)-like transcription factor genes to affect branch and tiller numbers (Zhu *et al.*, 2009; Wang *et al.*, 2015). Moreover, overexpression of OsmiR444a reduces the number of tillers through the repression of *OsMADS57* (Guo *et al.*, 2013). The *OsNAC2* locus, one of the target genes of miR164b, encodes a key transcription factor involved in rice development (Mao *et al.*, 2007; Fang *et al.*, 2014; Chen *et al.*, 2015).

Molecular characterization of genes controlling rice plant architecture and grain yield, and examination of their regulation will inform efforts to improve plant architecture and breed high-yield rice. In the present study, we overexpressed *OsNAC2* by mutating the OsmiR164b-binding site sequence. The resulting transgenic plants showed increased grain number and better plant architecture, which resulted in significantly improved grain yield per plant and overall rice yield in paddy fields. By manipulating *OsNAC2* expression, our work provides a new and practical approach for rice breeders to increase rice production.

## Materials and methods

### Plant and other experimental materials

Rice (*Oryza sativa* L.) plants were grown in a paddy field at the South China Agricultural University under natural conditions. Zhonghua 11 (ZH11), Yangyunjing 3, and Nipponbare were used as the wild type for analyses. At least 10 plants were used to measure agricultural traits for each experiment. *Escherichia coli* DH10B and *Agrobacterium tumefaciens* EHA105 were used for cloning and transformation experiments. pCAMBIA 1380 was used as the binary vector for *Agrobacterium*-mediated transformation.

For observing rice roots, rice seeds were germinated at room temperature. The seedlings were transplanted in Kimura B nutrient solution. The roots were separated one by one before being imaged on a scanner at the stage of flowering, and the roots were measured following the previously described method (Zhao *et al.*, 2004).

### Vector construction and genetic transformation

For overexpression of the miRNA, the precursor *OsmiRNA164b* was isolated by PCR using primers Ox164F and Ox164R. The PCR products were cloned into the *Pst*I– *Hin*dIII sites of pCAMBIA1380-Ubi containing the maize *Ubiquitin* promoter to construct the overexpression vector.

To overexpress *OsNAC2*, the ORF was amplified from cDNA using primers OxnacF and OxnacR. After subcloning and sequencing, the correct gene fragment was inserted into pCAMBIA1380-Ubi (driven by the *Ubiquitin* promoter).

To overexpress the miRNA164b-resistant *OsNAC2*, OxnacF and specific primers OxnacRm carrying the 5 bp mutations, OxnacFm carrying the 5 bp mutations, and OxnacR, respectively, were used to amplify two fragments of the *OsNAC2* sequence. After purifyication, the two fragments were mixed in equal molar ratios and used as the PCR template to amplify the full-length ORF of *OsNAC2* with the miRNA164b-resistant mutation, using OxnacF and OxnacR. The full-length ORF with the 5 bp mutations was then introduced into pCAMBIA1380-Ubi after sequencing.

To make the RNAi constructs, a fragment of *OsNAC2* was isolated by PCR using the primers RNAi1 and RNAi2 from the rice ZH11 genome into the *Eco*RI–*Hin*dIII sites and from immature panicle cDNA with primers RNAi2 and RNAi3 into the *Hin*dIII–*Sal*I sites of the cloning vector of pBluescript II. The RNAi construct was driven by the native promoter of *OsNAC2*. The *OsNAC2* promoter was amplified from the rice genome using the primers PRNAiF and PRNAiR, and cloned into the *Bam*HI–*Eco*RI sites of the cloning vector pBluescript II with RNAi fragments. After sequencing, the total fragments containing the native promoter and RNAi fragments were digested with *Bam*HI and *Sal*I, and cloned into the binary vector pCAMBIA1380. The cloning was performed following the previously described method (Li *et al.*, 2011; Zhou *et al.*, 2014). The primer sequences are listed in Supplementary Table S2 at *JXB* online.

Co-segregation analysis between the transgene and phenotype was performed using plants in the T_1_ and T_2_ generations to evaluate the effects of the transgenes.

### RNA extraction and quantitative real-time PCR (qRT-PCR)

RNA was isolated using the RNA extraction kit TRIzol Reagent (Invitrogen, USA) and quantified with DU730 (Beckman Coulter, Germany). Briefly, ~3 µg of total RNA was used to synthesize the first-strand cDNA. qRT-PCR was performed with the SYBR Premix ExTaq kit (TaKaRa) in a total volume of 20 µl on the BIO-RAD CFX connect following the manufacturer’s manual. Data were normalized to the internal rice *ubiquitin* (*UBI*) gene and the relative quantification method was used for data analysis. RT-PCR was performed with TaKaRa Ex Taq Hot Start Version following the manufacturer’s manual.

For quantification of mature OsmiR164b, stem–loop reverse transcription–quantitative PCR was performed as described (Chen *et al.*, 2005; Zhou *et al.*, 2012). All reactions were run in triplicate.

### Subcellular localization of OsNAC2

The *OsNAC2* cDNA sequence was cloned into the pUC18 vector with the coding sequence for enhanced green fluorescent protein (eGFP) under the control of the *Cauliflower mosaic virus* (CaMV) 35S promoter provided by Yang *et al.* (2014). Dehulled rice seeds of ZH11 were surface-sterilized using 1.5% sodium hypochlorite for 30 min, then germinated and cultured on half-strength Murashige and Skoog medium in the tissue culture room for ~7 d. Seedlings were grown hydroponically under natural light for 3 d. The protoplast isolation and transformation were performed following the method of Yang *et al.* (2014).

### Histological sectioning

The stems were collected from wild-type and transgenic plants at the flowering stage and sliced with a sharp blade. The sections were stained in 0.1% aqueous safranin for 3 min and washed in tap water for 1–3 min. The photos were taken with a light microscope.

### Measuring brown and milled rice percentages

For measuring the percentage of brown rice and milled rice, harvested paddy rice was completely dried. The processing was performed following the previously described method (Tan *et al.*, 2000). The chalkiness rate was determined following the method of Xu and Chen (2016).

### Sequence analysis

We analyzed the single nucleotide polymorphisms (SNPs) in the gene Os04g0460600 (including the promoter region) from the 3000 rice accessions of the 3000 rice genomes project, according to previously described methods (Li *et al.*, 2014; Alexandrov *et al.*, 2015).

### Accession numbers

Sequence data from this article can be found at the Rice Genome Annotation Project website or miRBase data libraries under the following accession numbers: *An-1* (LOC_Os04g28280), *DST* (LOC_Os03g57240), *HTD1* (LOC_Os04g46470), *HTD2* (LOC_Os03g10620), *SP1* (LOC_Os11g12740), *Ghd7* (LOC_Os07g15770), *DTH8* (LOC_Os08g07740), *IPA1* (LOC_Os08g39890), *DEP1* (LOC_Os09g26999), *OsNAC2* (LOC_Os04g38720), and *Ubi* (LOC_Os03g13170).

## Results

### *OsNAC2* is a target gene of miR164b

We cloned *OsNAC2* from a cDNA library of young rice panicles. We analyzed the subcellular localization of OsNAC2 using a transiently expressed GFP fusion in transformed rice protoplasts and found that OsNAC2–GFP localized to the nucleus (Supplementary Fig. S1). To overexpress OsNAC2, we cloned *OsNAC2* downstream of the maize *Ubi1* promoter and transformed this construct into the rice variety ZH11 to produce 11 independent transgenic lines (ZH11-*Ubi1*-overexpression *OsNAC2*, ZUOEN). However, no significant difference in morphology or agronomic traits was observed between the overexpression lines (lines ZUOEN 4 and ZUOEN 6) and wild-type ZH11 (Supplementary Fig. S2; Supplementary Table S1). RT-PCR analysis of the ZUOEN 4 and ZUOEN 6 lines revealed that the transcript level of *OsNAC2* was not significantly altered in the transgenic lines (Supplementary Fig. S2D), indicating that the *OsNAC2* mRNA levels might be post-transcriptionally regulated.

**Fig. 1.**
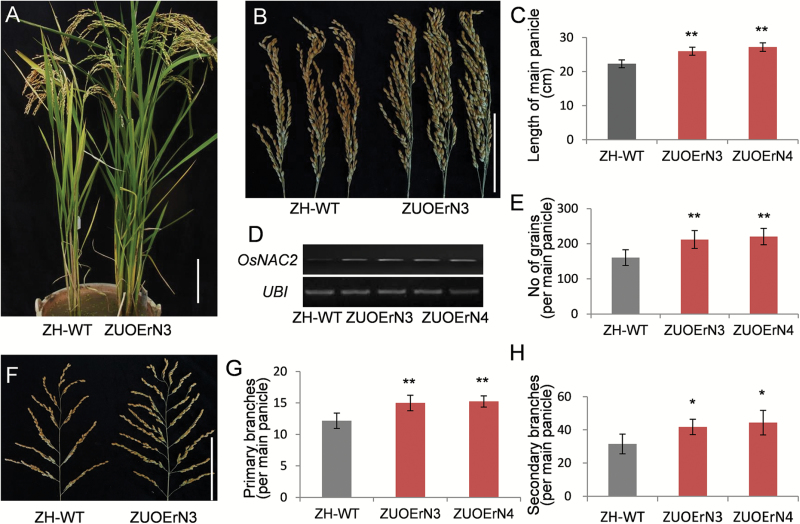
Morphologies of Zhonghua 11 and miR164b-resistant *OsNAC2* overexpression plants. (A–H) Characteristics of wild-type Zhonghua 11 (ZH-WT) and ZUOErN plants. (A) Morphologies. (B) Panicle morphologies. (C) Lengths of main panicle. (D) *OsNAC2* expression measured by RT-PCR. *Ubiquitin* was used as a loading control; (E) Grain numbers per main panicle. (F) Branch morphologies of the main panicle. (G) Number of primary branches. (H) Number of secondary branches. ZH-WT, wild-type Zhonghua 11. ZUOErN3 and ZUOErN4 are transgenic Zhonghua 11 plants overexpressing the miR164b-resistant *OsNAC2* driven by the maize *Ubiquitin1* (*Ubi1*) promoter. Means ±SD are given in (C), (E), (G), and (H) (*n*=10). *P<0.05; **P<0.01 (*t* test). Scale bars=10 cm.

A previous study demonstrated that *OsNAC2* is a target gene of miR164b (Fang *et al.*, 2014) (Supplementary Fig. S3). We further verified that *OsNAC2* is a target of miRNA164b by analyzing the sequence through the http://www. mirbase.org/ website. To verify the potential effect of miR164b binding on *OsNAC2* transcript levels, we introduced point mutations in the predicted miR164b-binding site without introducing any amino acid changes, and overexpressed this mutated version of *OsNAC2* using the *Ubi1* promoter (*Ubi*-overexpression miR164b-resisitant *OsNAC2*, *UOErN*) in ZH11 (Supplementary Fig. S3). Of the 14 independent transgenic lines we produced (ZH11-*UOErN*, ZUOErN), 11 displayed obvious phenotypic alterations such as vigorous growth (Fig. 1A) and enlarged main panicles (Fig. 1B, C). RT-PCR analysis showed increased *OsNAC2* transcript levels in lines ZUOErN3 and ZUOErN4 (Fig. 1D). These results suggest that increased levels of *OsNAC2* cause morphological changes in ZUOErN plants.

### Overexpression of miR164b-resistant *OsNAC2* increased grain number and yield

The ZUOErN plants showed better plant architecture compared with the wild-type (WT) ZH11 parental line. To test for any potential yield change in ZUOErN3 and ZUOErN4, we performed statistical analyses on single homozygous T_3_ plants. The grain yields for ZUOErN3 and ZUOErN4 in the early cropping season of 2011 were 22.07 ± 3.16 g and 20.18 ± 3.18 g per plant, respectively. Compared with grain yield of 11.43 ± 2.22 g per plant in ZH11, the production increased by 93.08% and 76.55% in ZUOErN3 and ZUOErN4, respectively (Table 1). Similarly, ZUOErN3 and ZUOErN4 produced 22.97 ± 2.37 g and 21.05 ± 3.81 g of grain per plant in the late cropping season, respectively, an increase of 78.75% and 63.81% compared with ZH11 (12.85 ± 3.28 g per plant) (Table 1). The grain yield per plant was consistently higher in ZUOErN3 and ZUOErN4 under various seasonal conditions, indicating that these lines had stable yields. Furthermore, the ZH11 and the transgenic lines showed no difference in the brown rice or milled rice percentage (Supplementary Fig. S5A, B). Moreover, we observed no obvious difference in grain phenotype among the WT, ZUOErN3, and ZUOErN plants (Supplementary Fig. S5D), although the chalkiness rate was higher in the transgenic lines than in the WT (Supplementary Fig. S5C).

**Table 1.** Yield test in a paddy field in several cropping seasons

Grain weight and plant season	WT	ZUOErN3	ZUOErN4
Grain weight per plant in 2011 early cropping season (g)	11.43 ± 2.22	22.07 ± 3.16***	20.18 ± 3.18***
Grain weight per plant in 2011 late cropping season (g)	12.85 ± 3.28	22.97 ± 2.37***	21.05 ± 3.81***
Total grain weight in 2012 early cropping season (g)	18 025.2	29 397.5	28 592.2

The planting density was 20 cm×20 cm. The area per plat was 2 m^2^ in 2011, 64 m^2^ in 2012. Data were calculated in block region with 40 plants under natural condition in 2011, Guangzhou China. Results represent means ±SD, ***P<0.001(*t*-test). Data were calculated for the total grain weight of 1600 plants for WT, ZUOErN3, and ZUOErN4, respectively. The rice was planted under natural conditions in 2012, Guangzhou China.

To evaluate the yield of ZUOErN plants in the field, in accordance with the approval document for field trials of genetically modified organisms from the Chinese Ministry of Agriculture (Nongji’an 2011-T013), field breeding and yield measurements were performed during the early cropping season of 2012 (Supplementary Fig. S4). Statistical analysis showed that, when compared with ZH11, the total yield of ZUOErN3 and ZUOErN4 increased by 63.06% and 58.62% in the field, respectively (Supplementary Fig. S4; Table 1), indicating that transgenic plants displayed increased yield potential under field conditions, which is consistent with the yield increase in single plants.

To investigate the reason for the increased grain number (Fig. 1E, F), we examined the main yield-related factors in the ZUOErN lines. The homozygous T_3_ ZUOErN3 and ZUOErN4 plants produced longer main panicles (25.97 ± 1.91 cm and 27.17 ± 1.64 cm, respectively), in comparison with ZH11 plants (22.27 ± 1.16 cm) (Fig. 1B, C). The number of primary branches per main panicle increased from 12.2 ± 1.21 in ZH11 to 15.0 ± 1.23 and 15.2 ± 0.89 in ZUOErN3 and ZUOErN4, respectively (*P*=5.045 × 10^–3^ and *P*=9.68 × 10^–4^) (Fig. 1G). Also, the number of secondary branches per main panicle increased by 32.7% and 40.6% in ZUOErN3 (41.75 ± 4.60) and ZUOErN4 (44.33 ± 7.41) plants (*P*=1.372 × 10^–2^ and *P*=1.422 × 10^–2^, respectively) (Fig. 1H). Moreover, the number of grains per main panicle significantly increased (*P*=3.192 × 10^–3^ and *P*=2.343 × 10^–3^, respectively) in ZUOErN3 (212.0 ± 25.5) and ZUOErN4 (220.3 ± 23.2) when compared with the values in ZH11 (160.3 ± 22.3) (Fig. 1E, F). These data indicate that the higher grain numbers in the overexpression lines are the result of increased panicle length and increased number of primary and secondary branches per panicle. However, we found no significant differences in 1000 grain weight or seed setting rates in ZUOErN3 and ZUOErN4 plants when compared with ZH11 plants (Supplementary Table S1). These data suggested that the increased yield in overexpression lines results from the increased numbers of grains produced.

Because the overexpression of *OsNAC2* caused an overall phenotypic change (Fig. 1A), we next analyzed effective tiller number. ZUOErN3 and ZUOErN4 plants produced an average of 9.93 ± 2.21 and 10.0 ± 2.49 productive tillers (*P*=3.468 × 10^–3^ and *P*=8.910 × 10^–4^), respectively, both of which are significantly higher than the productive tiller number of ZH11 plants (6.67 ± 1.11). In addition, the morphology of the tiller panicles resembled that of the main panicles of ZUOErN lines (Supplementary Fig. S6). In summary, ZUOErN plants displayed a moderate increase in productive tiller number, panicle length, branching number per panicle, grain number, and yield.

### The effects of *OsNAC2* on the stem, flag leaf, and root

**Fig. 2.**
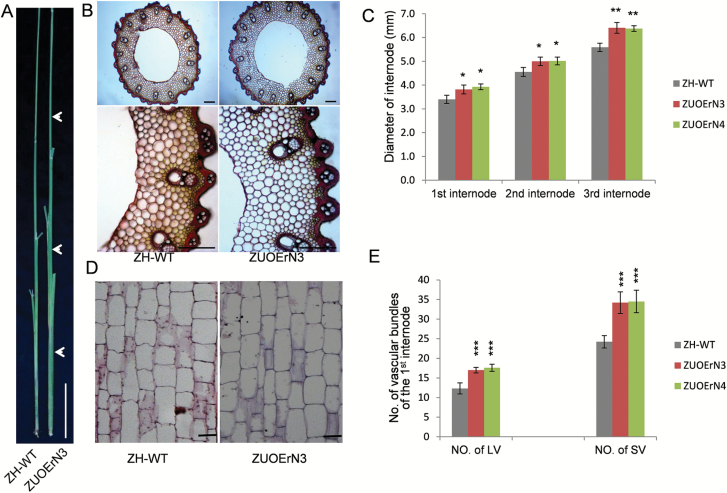
Internode phenotypes of ZUOErN plants. (A) Stem morphologies. The arrowheads indicated the elongated stem internodes. Scale bar=10 cm. (B) Cross-sections of the first elongated stem internodes. The photos below are enlarged images taken from the images above. Scale bars=200 μm. (C) Internode diameters. (D) Longitudinal sections of stem internodes. Scale bars=20 μm. (E) Numbers of large vascular bundles (LVs) and small vascular bundles (SVs) of the main stem at the first internode. ZH-WT, wild type Zhonghua 11. ZUOErN3 and ZUOErN4 are transgenic Zhonghua 11 plants overexpressing the miR164b-resistant *OsNAC2* driven by the maize *Ubiquitin1* promoter. Means ±SD are given in (C) and (E) (*n*=10). *P<0.05; **P<0.01 (*t*-test).

ZUOErN plants exhibited more vigorous growth and stronger stems than WT plants (Figs 1A, 2A), so we measured their agronomic traits related to the stem, flag leaf, and root. The stems of ZUOErN were thicker and stronger than those of WT plants (Figs 1A, 2A). Furthermore, the diameter of the first internode was 3.4 ± 0.17 mm in ZH11, whereas the diameter was 3.8 ± 0.19 mm in ZUOErN3 plants. The diameters of the second and third internodes of ZH11 plants were 4.6 ± 0.19 mm and 5.6 ± 0.17 mm, whereas they measured 5.0 ± 0.18 mm and 6.4 ± 0.22 mm, respectively, in ZUOErN3 plants (Fig. 2B, C).

Cross-sections of the stem internodes showed that the number of large vascular bundles in the first internode was significantly higher in ZUOErN3 and ZUOErN4 plants (17.0 ± 0.71 and 17.6 ± 0.89) (*P*=3.435 × 10^–6^ and *P*=7.583 × 10^–6^), when compared with ZH11 plants (12.3 ± 1.41) (Fig. 2E). The number of small vascular bundles was also significantly higher, 24.2 ± 1.58 in ZH11 plants in comparison with 34.2 ± 2.77 and 34.5 ± 2.87 in ZUOErN3 and ZUOErN4 plants (*P*=1.597 × 10^–6^ and *P*=1.462 × 10^–6^) (Fig. 2E). Transection showed that the stem had more cells, but cell size was similar to that of ZH11 (Fig. 2D). In addition, the number of large and small vascular bundles was higher in the middle part of the second and third internodes in the ZUOErN plants (Supplementary Fig. S9).

The average height of ZUOErN3 and ZUOErN4 plants showed a significant increase, 10.6% and 10.7% (*P*=2.768 × 10^–2^ and *P*=3.529 × 10^–2^), respectively, when compared with the 98.0 ± 3.19 cm height of ZH11 plants (Supplementary Table S1). Longitudinal sections showed that the longer stems of ZUOErN3 plants are due to elongated cells, compared with ZH11 plants (Fig. 2D).

**Fig. 3.**
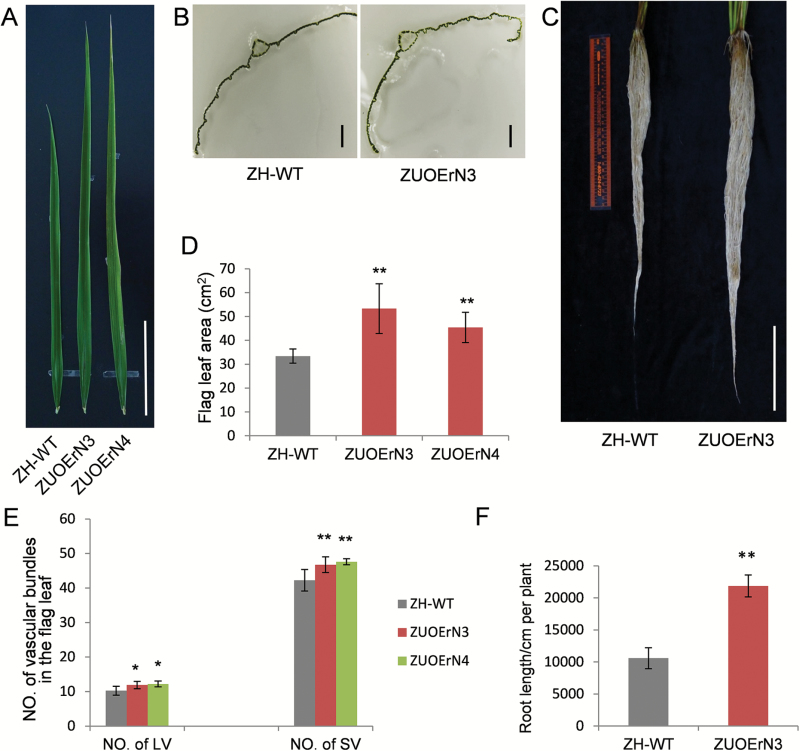
Flag leaf and root phenotypes of ZUOErN plants. (A) Flag leaf morphologies. Scale bar=10 cm. (B) Cross-sections of flag leaves. Scale bars=1 cm. (C) Root morphologies of water-cultured ZH-WT and ZUOErN3 plants at the flowering stage. Scale bar=10 cm. (D) Area of mature flag leaves. (E) Numbers of large and small vascular bundles in the middle section of flag leaves. (F) Total root lengths per plant in the flowering stage. ZH-WT, wild-type Zhonghua 11. ZUOErN3 and ZUOErN4 are transgenic Zhonghua 11 plants overexpressing the miR164b-resistant *OsNAC2* driven by the maize *Ubiquitin1* promoter. Means ±SD are given in (D) (*n*=10) and (F) (*n*=4). *P<0.05; **P<0.01 (*t*-test).

Flag leaves are the main source organs that determine grain yield potential (Tan *et al.*, 2012). The ZH11 plants had a flag leaf area of 33.40 ± 2.96 cm^2^; the flag leaf areas of ZUOErN3 and ZUOErN4 were 53.31 ± 10.42 cm^2^ and 45.41 ± 6.36 cm^2^ (*P*=3.089 × 10^–4^ and *P*=1.276 × 10^–3^), respectively (Fig. 3A, D). More importantly, the flag leaf angles did not change noticeably in ZUOErN3 and ZUOErN4 plants when compared with ZH11 (Fig. 1A). These results suggested that the ZUOErN plants may have a higher photosynthetic rate. In-depth characterization indicated that in the central part of the flag leaves, the number of large vascular bundles was significantly higher in ZUOErN3 and ZUOErN4 plants (11.89 ± 1.05 and 12.20 ± 0.84, respectively) when compared with the average of 10.25 ± 1.28 in ZH11 plants (*P*=1.115 × 10^–2^ and *P*=1.208 × 10^–2^) (Fig. 3B, E). There were more small vascular bundles in ZUOErN3 and ZUOErN4 plants (46.75 ± 2.25 and 47.60 ± 0.89, respectively) than in ZH11 plants (42.25 ± 3.11) (*P*=5.076 × 10^–3^ and *P*=3.491 × 10^–3^) (Fig. 3B, E). The increased number of vascular bundles in the overexpression lines may facilitate the transport of photosynthetic products. Moreover, the ZUOErN plants showed much better developed vascular systems and mechanical tissues (Fig. 2B, E), which is crucial for high yield in rice.

The transgenic plants had longer root systems when compared with ZH11 plants in the flowering period (Fig. 3C). The total length of the root system in the ZUOErN3 plants was 21 853 ± 1719 cm, representing a 106.6% increase in comparison with the 10 578 ± 1643 cm of ZH11 per plant (*P*=1.200 × 10^–3^) (Fig. 3F). Denser and longer roots in transgenic rice plants may facilitate the absorption of water and nutrients, therefore contributing to a higher grain yield. In summary, these results suggested that the structural changes in the leaves, stems, and roots in the overexpression lines may facilitate the improved grain number and enhanced yield.

### Expression of genes involved in grain number in ZUOErN plants

Plants overexpressing *OsNAC2* showed increased grain number and higher yield; we therefore measured several yield-related genes, such as *IPA1*, *DEP1*, and *Ghd7* in ZUOErN3 and ZUOErN4 plants. The qRT-PCR analysis revealed a significant up-regulation of *IPA1* in ZUOErN3 and ZUOErN4 transgenic plants (Supplementary Fig. S7A). Previous studies have demonstrated that IPA1 increases grain number through the up-regulation of *DEP1* (Lu *et al.*, 2013). We therefore measured the expression levels of *DEP1* as well and found that it was up-regulated by >4-fold in *OsNAC2*-overexpressing lines (Supplementary Fig. S7B). Furthermore, we found that the expression level of *DTH8* was reduced by 24.5% and 29.0% in ZUOErN3 and ZUOErN4 plants, respectively, and the expression of *Ghd7* was reduced by 36.3% and 39.8%, respectively (Supplementary Fig. S8). qRT-PCR analysis of *OsNAC2*-overexpressing lines showed no obvious alteration in the expression level of *An-1*, *DST*, *HTD1*, *HTD2*, and *SP1* (Supplementary Fig. S8) that are related to grain number. These results suggested that *OsNAC2* may control grain number and plant architecture through an *IPA1–DEP1*-related pathway.

### *OsNAC2*-RNAi plants have small panicles

**Fig. 4.**
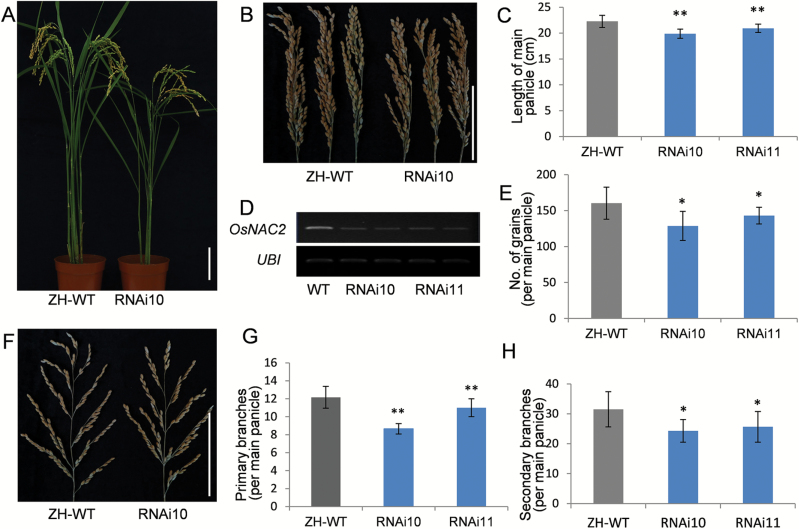
Phenotypes of *OsNAC2* RNAi plants. (A) Morphologies. (B) Panicle morphologies. (C) Panicle length. (D) *OsNAC2* expression in ZH-WT and RNAi plants detected by RT-PCR. (E) Grain numbers per main panicle. (F) Branch morphologies of the main panicle. (G) Number of primary branches. (H) Number of secondary branches. WT and ZH-WT, wild-type Zhonghua 11. RNAi10 and RNAi11 are transgenic Zhonghua 11 plants with the *OsNAC2* RNAi vector driven by its native promoter. Means ±SD are given in (C), (E), (G), and (H) (*n*=10). *P<0.05; **P<0.01 (*t*-test). Scale bars=10 cm.

To examine the effect of decreased *OsNAC2* expression, we constructed an RNAi vector against *OsNAC2* driven by the native *OsNAC2* promoter (Li *et al.*, 2011). We transformed the construct into ZH11 plants and obtained 12 independent RNAi lines. Seven of these RNAi lines displayed a stunted phenotype (Fig. 4A; Supplementary Table S1) with panicles shorter than those of ZH11 plants (Fig. 4B, C). RT-PCR indicated a significant reduction of the *OsNAC2* expression level in the RNAi plants (Fig. 4D). When compared with ZH11 plants, the panicle length of RNAi10 and 11 lines was 10.68% and 6.11% shorter (Fig. 4C), and the grain number was reduced by 19.71% and 10.79%, respectively (Fig. 4E, F). In addition, the number of both primary and secondary branches of the main panicles was reduced in the RNAi plants (Fig. 4G, H). However, no significant differences in tiller number, 1000 grain weight, or seed setting rate were observed in RNAi plants when compared with the WT (Supplementary Table S1). Taken together, the overexpression and RNAi experiments indicate that *OsNAC2* controls multiple yield-related traits in rice plants, including panicle length, branch number, and grain number.

### Overexpression of *OsmiR164b* leads to small panicles

**Fig. 5.**
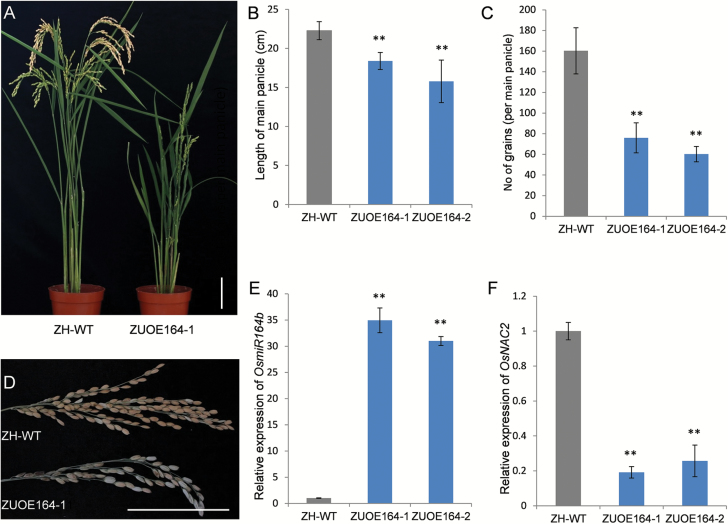
Phenotypes of plants overexpressing *OsmiR164b*. (A) Morphologies in the mature stage. (B) Length of main panicles. (C) Grain number per main panicle. (D) Morphologies of main panicles. (E) OsmiR164b expression detected by qRT-PCR. (F) *OsNAC2* expression detected by qRT-PCR. ZH-WT, wild-type Zhonghua 11. ZUOE164-1 and ZUOE164-2 are transgenic Zhonghua 11 plants overexpressing *OsmiR164b* driven by the maize *Ubiquitin 1* promoter. Means ±SD are given in (C), (E), (G), and (H) (*n*=10). *P<0.05; **P<0.01 (*t*-test). Scale bars=10 cm.

Our results indicated that miR164b regulates *OsNAC2* (Supplementary Figs S2, S3). To confirm further the regulation of *OsNAC2* by miR164b and its effects on yield-related traits, an overexpression construct containing *OsmiR164b* driven by the maize *Ubi1* promoter was generated and introduced into ZH11 plants (Zhonghua11-*Ubi1*-overexpression OsmiR164b, ZUOE164). Of the 18 transgenic lines obtained, 17 exhibited dwarfism and short panicles (Fig. 5A, D). The main panicle length was 18.38 ± 1.09 cm for ZUOE164-1 and 15.78 ± 2.73 cm for ZUOE164-2 plants, both of which significantly differed from the panicle length of ZH11 plants (Fig. 5B). The grain numbers also decreased from 160.3 ± 22.3 in ZH11 plants to 76.0 ± 14.5 and 60.3 ± 7.4 in ZUOE164-1 and -2 plants, respectively (Fig. 5C). The ZUOE164-1 and -2 plants had fewer primary and secondary branches (Supplementary Fig. S10). Using qRT-PCR, we found that miR164b expression levels increased in ZUOE164 plants (Fig. 5E) whereas *OsNAC2* transcript levels decreased (Fig. 5F). Down-regulation of *OsNAC2* leads to smaller panicles and fewer grains in plants overexpressing OsmiR164b or expressing *OsNAC2*-RNAi, which demonstrated that miR164b regulates panicle length and grain number by modulating *OsNAC2* transcript levels.

### The binding site for miR164 is highly conserved

Our study revealed that the overexpression of *OsNAC2* with a mutated miRNA-binding site significantly enhanced the yield potential of rice plants. To determine whether a similar mutation had occurred under natural conditions or during domestication, we obtained the sequences of *OsNAC2* from the genome sequences of 3000 rice accessions (Li *et al.*, 2014). We identified 33 SNPs in the promoter and coding regions of *OsNAC2* among 3000 rice varieties (Li *et al.*, 2014; Alexandrov *et al.*, 2015). However, we detected no mutation in the binding site of miR164b in *OsNAC2* between positions 22 995 991 and 22 996 010 of chromosome 4 (Supplementary Table S3), suggesting that this sequence is evolutionarily conserved in rice. This observation explains why yield-enhancing alleles of *OsNAC2* have not been found in rice cultivars.

### Potential application of *OsNAC2* in high-yield breeding

**Fig. 6.**
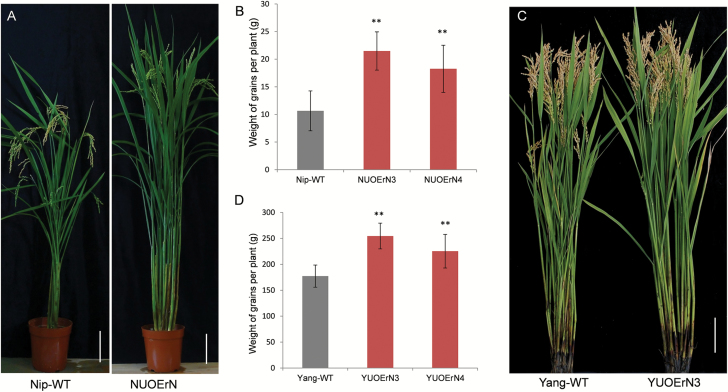
Phenotypes of plants overexpressing miR164b-resistant *OsNAC2* in Nipponbare (NUOErN) and Yangyujing 3 (YUOErN) backgrounds. (A and C) Morphologies at the mature stage. Scale bars=10 cm. (B and D) Single plant yield. Nip-WT, Nipponbare wild type. NUOErN are transformed from ZUOErN plants. Yang-WT, Yangyujing 3 wild type. YUOErN are transformed from ZUOErN plants. Means ±SD are given in (B) and (D) (*n*=20). **P<0.01 (*t*-test). Nip-WT and NUOErNs were planted in 2014 in Guangzhou, China. Yang-WT and YUOErN were planted in 2015 in Sanya, China.

To evaluate the function and potential application of *OsNAC2* in other rice varieties, we transferred the miR164b-resistant *OsNAC2* transgene into Nipponbare (Nipponbare-*UOErN*, NUOErN) by backcrossing with recipient lines. The obtained NUOErN3 and NUOErN4 lines showed IPA traits such as increased tiller number, when cultivated in Guangzhou, Guangdong province, China (Fig. 6A). The grain weight per plant significantly increased from 10.36 ± 3.60 g in control plants to 21.49 ± 3.46 g and 18.27 ± 4.26 g in NUOErN3 and NUOErN4 plants, respectively (Fig. 6B). These results indicate that overexpression of *OsNAC2* has a similar effect on increasing grain yield in the Nipponbare rice variety.

Yangyujing 3 is a widely used new rice variety with high yield and good grain quality. We produced Yangyujing 3 plants containing the miR164b-resistant *OsNAC2* transgene by backcrossing with recipient lines (Yangyujing3-*UOErN*, YUOErN) and grew the plants in winter 2015 in Sanya, Hainan province, China. Statistical analysis of agronomic traits of these plants showed that YUOErN3 and YUOErN4 lines had improved architecture and enlarged panicles (Fig. 6C). The grain weight per plant also increased from 41.8 ± 10.01 g in control plants to 66.3 ± 12.44 g and 55.46 ± 9.10 g in YUOErN3 and YUOErN4 plants, respectively (*P*=8.743 × 10^–3^ and *P*=3.866 × 10^–2^) (Fig. 6D). Taking these observations together, we conclude that expression of an miRNA-resistant *OsNAC2* can be used to improve the yield in rice.

## Discussion

Improving grain yield has been a primary goal for rice breeding. At the end of the last century, Japanese scientists and researchers at the International Rice Research Institute (IRRI) proposed the concept of super-high yield breeding and super rice varieties, and put these concepts into practice (Peng *et al.*, 2008; Xu and Chen, 2016). In 1996, the Chinese Ministry of Agriculture initiated the Super Rice Project, aiming to improve rice yield by combining heterosis between subspecies and the establishment of ideal plant architecture (Qian *et al.*, 2016; Xu and Chen, 2016). Recently, several super hybrid rice combinations have been cultivated by breeders, providing concrete evidence for the utility of this theory (Qian *et al.*, 2016). Ideotype breeding or ideal plant architecture includes low tiller number, fewer or no unproductive tillers, high grain number per panicle, and thick and sturdy stems, as proposed by the IRRI (Peng *et al.*, 2008; Miura *et al.*, 2010; Lu *et al.*, 2013.

*IPA1/OsSPL14* affects these features and was identified a few years ago; an SNP in the coding region of *IPA1* affects miR156 targeting and confers an ideal plant architecture (Jiao *et al.*, 2010; Miura *et al.*, 2010). IPA1 is regulated at the transcriptional and post-transcriptional levels (Lu *et al.*, 2013; Wang *et al.*, 2017; Zhang *et al.*, 2017). IPA1 may be ‘a new green revolution gene’, as IPA-mediated traits can be fine-tuned by manipulation of *IPA1* expression, which may further improve yield (Wang and Wang, 2017; Zhang *et al.*, 2017). In this study, the *OsNAC2* overexpression plants had thicker stems, longer panicles, and increased grain number, which are characteristic of IPA, and showed significantly increased yield under field conditions. The expression level of both *IPA1* and *DEP1* was increased in ZUOErN plants. Our findings show that *OsNAC2* is an important regulator of rice architecture and could be useful for breeding new high-yield varieties.

In this study, we provided evidence that *OsNAC2* is a target gene of miR164b. Overexpression of miR164b-resistant *OsNAC2* greatly enhanced grain number and rice yield. When *OsNAC2* driven by the *Ubi* promoter was introduced in rice, there was no significant difference between the transgenic and the WT plants. However, when point mutations were introduced in the binding site sequence without any amino acid changes, plants transformed with the mutated *OsNAC2* gene (ZUOErN) displayed improved grain number and enhanced grain yield in several planting seasons. An increased transcript level of *OsNAC2* was observed in the ZUOErN lines. Furthermore, in OsmiR164b overexpression plants and OsNAC2-RNAi plants, the level of *OsNAC2* transcript was decreased, and the panicle length and grain number were decreased as well. We therefore speculate that the expression of *OsNAC2* is a critical factor for determination of grain number.

Traditional breeding programs largely rely on natural mutations and long-term artificial selection. Many agronomic traits are commercially important and have been characterized at the molecular level (Jin *et al.*, 2008; Tan *et al.*, 2008; Xue *et al.*, 2008; Huang *et al.*, 2009; Jiao *et al.*, 2010; Li *et al.*, 2010; Miura *et al.*, 2010; Qian *et al.*, 2016; Si *et al.*, 2016). Naturally occurring yield-related alleles that affect miRNA-related functions have been identified for only a few genes (Jiao *et al.*, 2010; Wang *et al.*, 2012; Che *et al.*, 2015; Duan *et al.*, 2015; Gao *et al.*, 2015). Using a whole-genome alignment of 3000 rice accessions (Li *et al.*, 2014), we found that the binding site for miR164b in *OsNAC2* showed a high degree of conservation, suggesting that natural variation in this site will not prove useful for improvement of rice yields using traditional breeding methods. Therefore, novel methods to create new rice varieties with higher, more stable yields are required. In this study, rice plants with significantly increased yield were created by introducing mutations into the miR164b-binding site of *OsNAC2*. Our research also provides a starting point for future investigation and applications of similar genes.

The *OsNAC2* gene appears to play an important role in rice development. However, different functions have been ascribed to OsNAC2 in different studies. *Ostill*, a CaMV35S promoter activation-tagging mutant, in which the expression of *OsNAC2* is significantly up-regulated, led to wider tiller angles and reduced plant height (Mao *et al.*, 2007). However, when *OsNAC2* (*OMTN2*) was overexpressed, driven by the *Ubi1* promoter, the transgenic rice plants displayed no altered phenotypes, with the exception of hypersensitivity to drought at the reproductive stage (Fang *et al.*, 2014). In addition, Chen *et al.* (2015) found that plants overexpressing *OsNAC2* under the control of the CaMV35S promoter were shorter and that *OsNAC2* regulates genes that are involved in the gibberellin biosynthetic pathway. In the present study, we overexpressed *OsNAC2* with the native miR164-binding site under the control of the *Ubi1* promoter, and found that neither *OsNAC2* expression nor plant morphology was altered in transgenic plants. Nevertheless, plants expressing miR164-resistant *OsNAC2* driven by the *Ubi1* promoter showed improved plant architecture. Taken together, these results indicate that the function of *OsNAC2* may be related to different expression patterns, or different insertion positions in the chromosome.

## Supplementary data

Supplementary data are available at *JXB* online.

Table S1. The agronomic traits of transgenic and wild-type plants.

Table S2. Primers used for RT-PCR and plasmid construction.

Table S3. The SNP type of each rice accession from ‘The 3000 rice genomes project’ in the gene *OsNAC2.*

Fig. S1. Nuclear localization of the OsNAC2–GFP fusion protein in rice leaf sheath protoplasts.

Fig. S2. Morphologies of Zhonghua 11 and *OsNAC2* overexpression plants.

Fig. S3. Location of the OsmiR164b-binding site in the *OsNAC2* nucleotide sequence.

Fig. S4. Phenotype of ZUOErN transgenic lines in field conditions.

Fig. S5. Main processing quality traits in ZH-WT, ZUOErN3, and ZUOErN4 transgenic plants.

Fig. S6. Uniformity of panicles from single plants of ZH-WT and ZUOErN3 plants.

Fig. S7. Up-regulation of *IPA1* and *DEP1* in ZUOErN transgenic plants.

Fig. S8. Expression level of grain number-related genes in ZUOErN transgenic plants.

Fig. S9. Number of large and small vascular bundles in second and third internodes in the stem.

Fig. S10. Branch number per main panicle of OsmiR164b overexpression plants.

Supplementary Figures Tables

Supplementary Tables 3
