# Supplementary material for: Overexpression of miR164b-resistant OsNAC2 improves plant architecture and grain yield in rice
Source: J Exp Bot. 2018 Jan 20;69(7):1533–43. doi: 10.1093/jxb/ery017 (PMC5888996; doi:10.1093/jxb/ery017)
Supplement: Supplementary Figures Tables [file ery017_suppl_fig_s1_s10_tab_s1_s2.pdf]

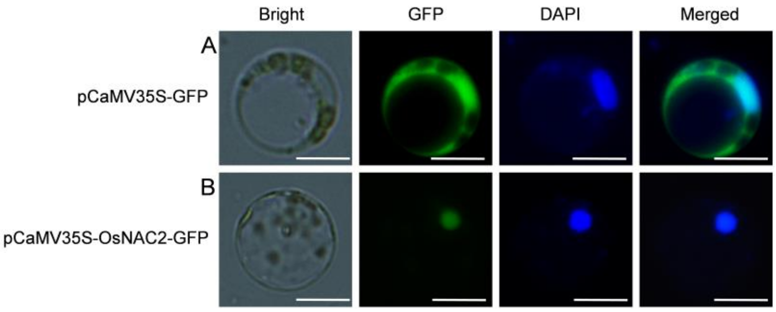

Supplemental Figure Lengends:

Supplemental Fig. S1. Nuclear localization of the OsNAC2-GFP fusion protein in rice leaf sheath protoplasts.

The pCaMV35S-GFP (A) and pCaMV35S-OsNAC2-GFP (B) constructs were transformed into rice leaf sheath protoplasts. The pCaMV35S-OsNAC2-GFP (B) GFP signal (green) localized in the nucleus. The transformed protoplasts were subsequently stained with DAPI for 5 minutes to localize the nuclei. Scale bars, 10 μm.

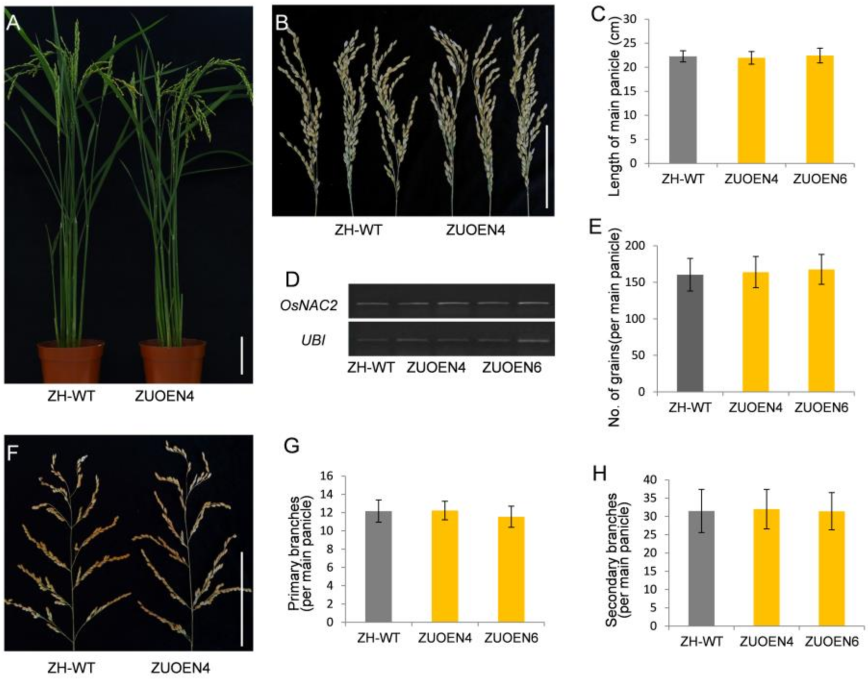

Supplemental Fig. S2. Morphologies of Zhonghua 11 and *OsNAC2* overexpression plants. (A) – (H), characteristics of wild-type Zhonghua 11 (WT-ZH) and ZUOEN plants.

(A) Morphologies. (B) Panicle morphologies. (C) Lengths of main panicle.

(D) *OsNAC2* expression in WT-ZH and ZUOEN plants detected by RT-PCR

(E) Grains per main panicle. (F) Branch morphologies of the main panicle. (G) Number of primary branches. (H) Number of secondary branches.

WT-ZH: wild-type Zhonghua 11, ZUOEN4 and ZUOEN6 are transgenic Zhonghua 11 plants with *OsNAC2* driven by the maize *Ubiquitin1* (*Ubi1*) promoter.

Means ± SD are given in C, E, G, and H (*n*=10). Scale bars, 10 cm.

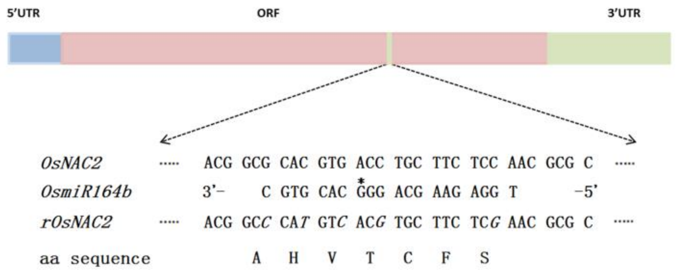

Supplemental Fig. S3. Location of the OsmiR164b binding site in the *OsNAC2* nucleotide sequence.

Breakout shows *OsNAC2* and *OsrNAC2* mRNA sequence and the corresponding OsmiR164b sequence. The mismatched base between OsmiR164b and wild-type *OsNAC2* is shown in asterisk Introduced point mutations used to disrupt OsmiR164b and *OsNAC2* are shown in italics.

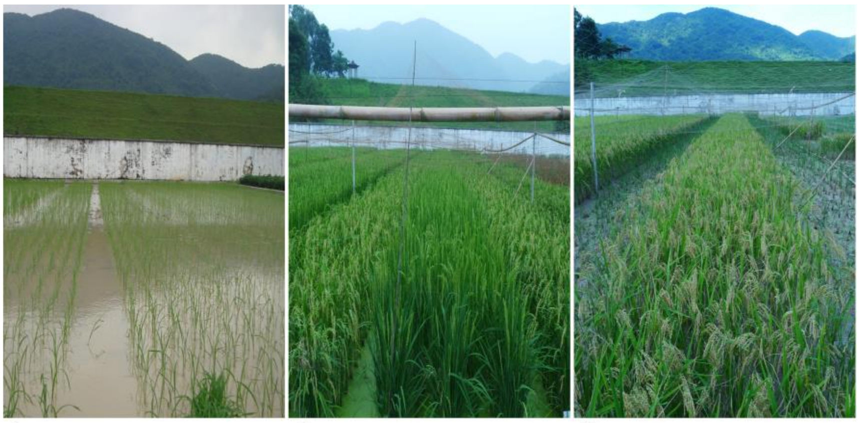

A B C

Supplemental Fig. S4. Phenotype of ZUOErN transgenic lines in field conditions in the seedling stage (A), heading stage (B), and mature stage (C).

The planting density was 20 cm × 20 cm. The area per plat was 64 m^2 in 2012, Guangzhou China.

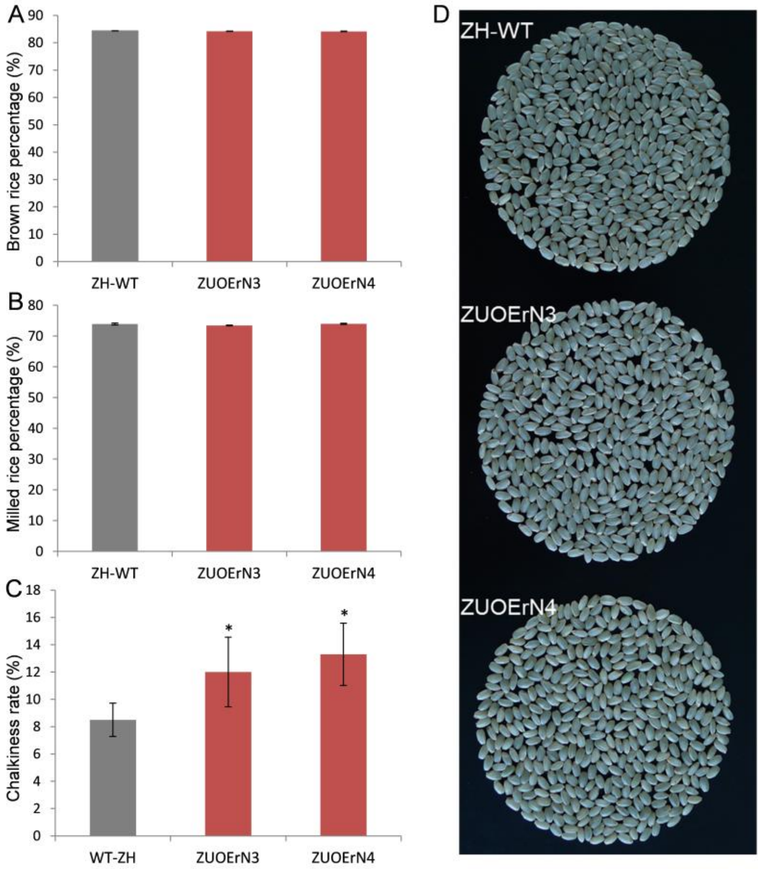

Supplemental Fig. S5. Main processing quality traits in WT-ZH, ZUOErN3, and ZUOErN4 transgenic plants.

(A) Brown rice percentage. (B) Milled rice percentage. (C) Chalkiness rate. (D) Grains Phenotype. WT-ZH: wild type Zhonghua 11. ZUOErN3 and ZUOErN4 are transgenic Zhonghua 11 plants overexpressing the miR164b-resistant *OsNAC2* driven by the maize *Ubiquitin1* (*Ubi1*) promoter. Means ± SD are given (*n*=3) in A and B; While Means ± SD are given (*n*=5) in C, Sample size is 100 grains.

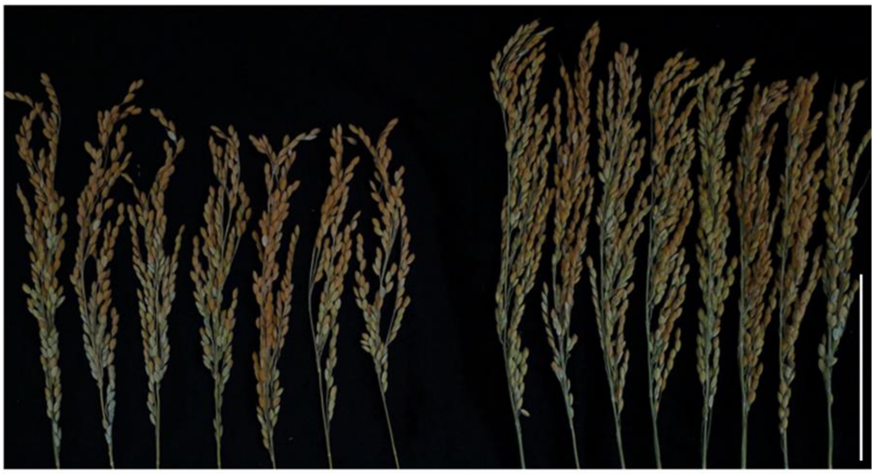

Supplemental Fig. S6. Uniformity of panicles from single plants of WT-ZH and ZUOErN3 plants.

Scale bar, 10 cm.

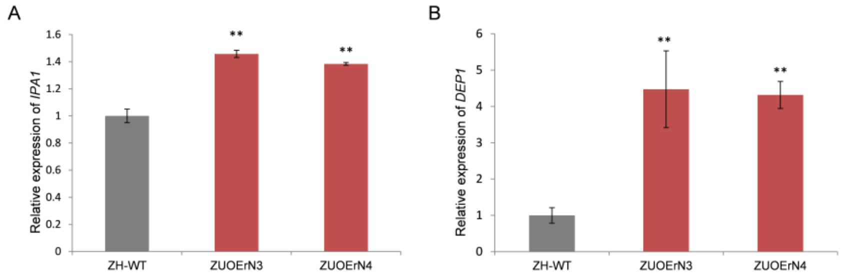

Supplemental Fig. S7. Upregulation of IPA1 and DEP1 in ZUOErN transgenic plants.

Expression of *IPA1* and *DEP1* was analyzed by qRT-PCR analysis. Every analysis was repeated 3 times.

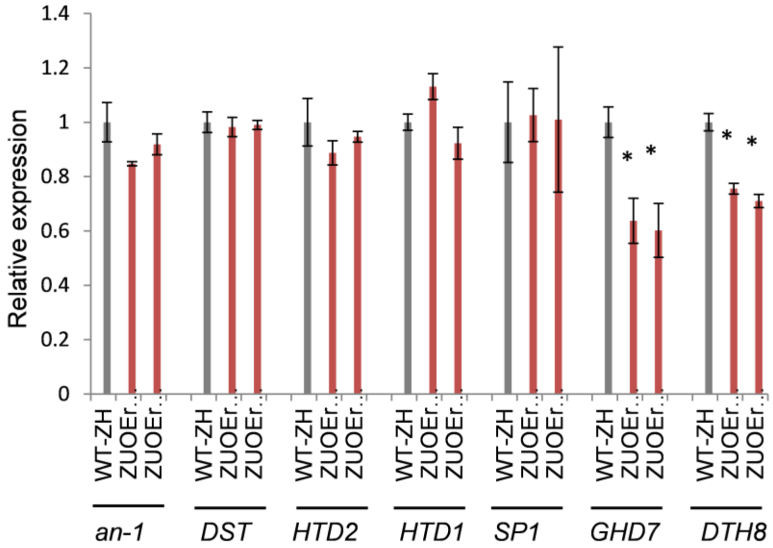

Supplemental Fig. S8. Expression levels of grain number-related genes in ZUOErN transgenic plants.

**P<0.01 (*t* test)

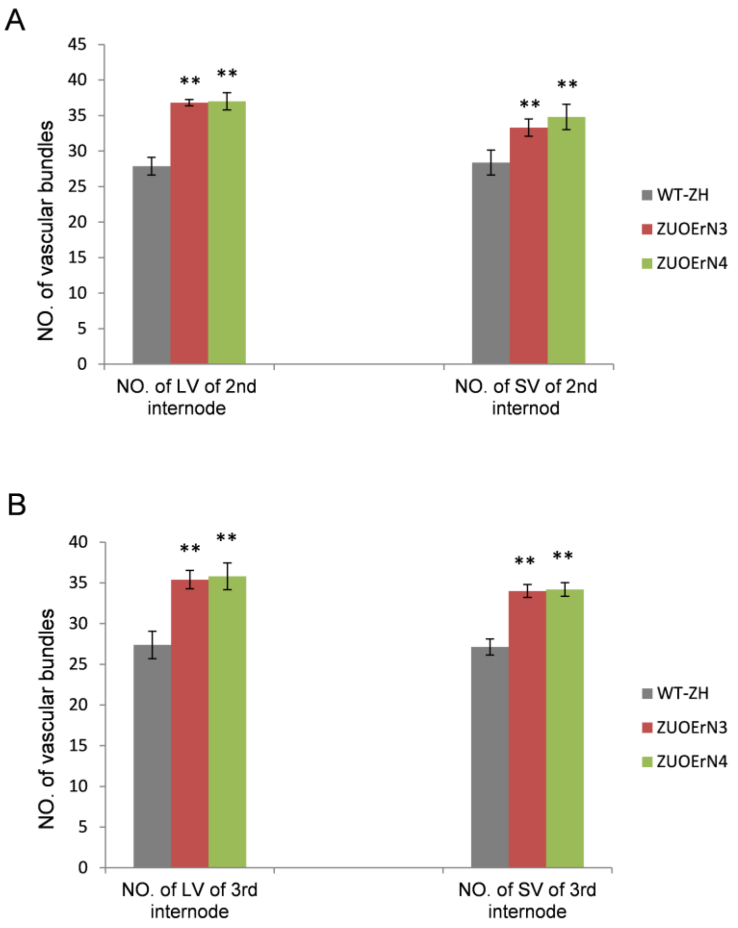

Supplemental Fig. S9. Number of large and small vascular bundles in second and third internodes in the stem.

(A) Second internode. (B) Third internode.

WT-ZH: wild type Zhonghua 11. ZUOErN3 and ZUOErN4 are transgenic Zhonghua 11 plants overexpressing the miR164b-resistant *OsNAC2* driven by the maize *Ubiquitin1* (*Ubi1*) promoter.

Means ± SD are given in A and B (n=10). **P<0.01 (t test).

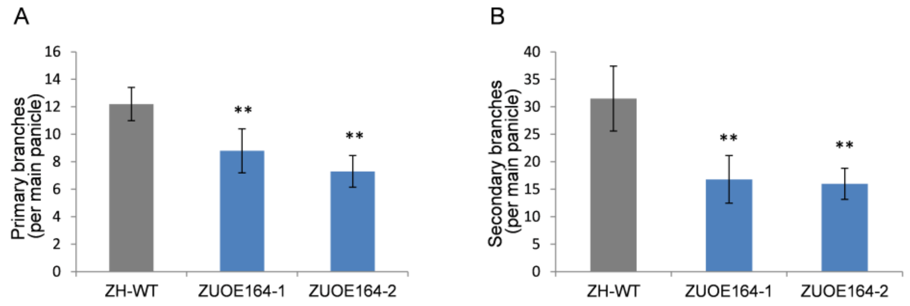

Supplemental Fig. S10. Branch number per main panicle of OsmiR164b overexpression plants.

Means ± SD are given in A and B (*n*=10). ***P*<0.01 (*t* test).

Supplementary Table S1. The agronomic traits of transgenic plants and wild type

Plant line	Plant height (cm)	Effective tiller number	Setting percentage of main panicle (%)	1 000 grain weight (g)
WT	98.0±3.19	6.7±1.11	89.7±2.9	27.65±0.14
ZUOEN4	97.0±3.44	6.7±2.00	88.8±4.1	27.45±0.30
ZUOEN6	98.6±3.43	7.1±1.84	89.8±6.1	27.58±0.17
ZUOErN3	103.5±4.69*	9.9±2.21**	89.7±2.9	27.65±0.21
ZUOErN4	104.7±3.35*	10.0±2.49***	89.8±5.3	27.98±0.33
RNAi10	80.2±3.87**	7.4±1.00	91.0±2.0	27.55±0.19
RNAi11	82.3±3.67*	6.5±0.80	87.9±6.6	27.62±0.11

The data were indicated using average ± SD (standard deviation). The sample size for traits of plant height, effective tiller number and setting percentage of main panicle was 10, while 3 for 1 000 grain weight. Asterisks indicate the significance of differences between each of the transgenic lines and WT detected by the method of independent samples *t*-test: *, P<0.05; **, P<0.01; ***, P<0.001.

Supplementary Table S2. Primers used for RT-PCR and plasmid construction

Primer	Sequence (5' to 3')	Purpose
Ox164F	AAACTGCAGAAGCTAAGAATCTCGTCAGG	Overexpression vector construction
Ox164R	AAAGCTTAATCAGATCAGGTGAATGAAT	
OxnacF	AAAAAAAGCTTACAACGATTTCCTCTTGTCACC	Overexpression vector construction
OxnacR	AAAAAACTAGTGTAGATGCCTCGATCGCGATCT	
OxnacFm	GCCCATGTCACGTGCTTCTCGAACGCGCTGGAGGGC CAGTTCTT	OsmiR164b-resistant overexpression

OxnacRm	TCGAGAAGCACGTGACATGGGCCGTCGTCGCCGGG TCGACGG	vector construction
PnacF	AAGGATCCCCCCATCAGCAGTATTCTCT	*OsNAC2* RNAi vector construction
PnacR	CCGATATCTGCTGCCTAAGCTAGCTAGC	
RNAi1	AAGAATTCGCTAGCTTAGGCAGCAATGG	
RNAi2	AA AAGCTTTTCTCCCCCATCTTCGCCAGA	
RNAi3	AAGTCGACGCTAGCTTAGGCAGCAATGG	
gfpF	AAGGATCCGCATGGAGCAGCATCAGGGCCAG	Transient fusion eGFP expression vector
gfpR	AA AAGCTTGTAGCCCCATAGCGCGGCCTC	
qAn-1F	CGGCGACCTCTCCTTCTGTA	Real-time PCR for *OsAn-1*
qAn-1R	TGCTGCTGGTGATGCTGATG	
qDSTF	ATCCAAGAAGGCAAGGTCAATC	Real-time PCR for *OsDST*
qDSTR	ACACACGAGGAGGAATTGGAA	
qHTD1F	ACCTCGTCCAGAAGCGTGAGT	Real-time PCR for *OsHTD1*
qHTD1R	AGGCCCAGTCGTGGATCA	
qHTD2F	GGTTCTTGAACGACAGCGACTA	Real-time PCR for *OsHTD2*
qHTD2R	CGTCGAACACCTGCTGTATCTC	
qSP1F	CGGTAACCAAGAGGAAACAAGTG	Real-time PCR for *OsSP1*
qSP1R	CACCACGCACAGTAGCACCTT	
qGhd7F	AGGTGCTACGAGAAGCAAATCC	Real-time PCR for *OsGhd7*
qGhd7R	GGCGAAGCGACCTCTCACTC	
qDTH8F	CAGGAGTGCGTGTCGGAGTT	Real-time PCR for *OsDTH8*
qDTH8R	GGTCGTCGCCGTTGATGGT	
qIPA1F	TGCATTCCAAGGCTCCCCGC	Real-time PCR for *OsIPA1*
qIPA1R	TGCGGCAGCTGCGTTTTCCT	
qDEP1F	GCGAGATCACGTTCCTCAAG	Real-time PCR for *OsDEP1*
qDEP1R	TGCAGTTTGGCTTACAGCAT	
qOsNAC2 F	GTCGTCCGTCGTCCTCCCTC	RT-PCR for *OsNAC2*
qOsNAC2 R	CTGCCGTCGGGTTAAAGAACTG	
qUbiF	AACCAGCTGAGGCCCAAGA	Real-time PCR for *OsUBQ*
qUbiR	ACGATTGATTTAACCAGTCCATGA	
qOs164bF	TGGAGAAGCAGGGCACG	Real-time PCR for *OsmiR164b*
qOs164bR	GTGCAGGGTCCGAGGTATTC	
RT-164b	GTCGTATCCAGTGCAGGGTCCGAGGTATTCGCACTG GATACGACTGCAC	Reverse transcript for *OsmiR164b*

Supplementary Table S3. The SNP type of each rice accessions from 'The 3,000 rice genomes project' in the gene *OsNAC2*
